# Reference genome of *Calochortus tolmiei* Hook. & Arn. (Liliaceae), a cat's ear mariposa lily

**DOI:** 10.1093/g3journal/jkaf008

**Published:** 2025-01-20

**Authors:** Jacob B Landis, Julianna J Harden, Evan Eifler, Gretta L Buttelman, Adriana I Hernández, Thomas J Givnish, Susan R Strickler, Chelsea D Specht

**Affiliations:** School of Integrative Plant Science, Section of Plant Biology and the L.H. Bailey Hortorium, Cornell University, Ithaca, NY 14853, USA; School of Integrative Plant Science, Section of Plant Biology and the L.H. Bailey Hortorium, Cornell University, Ithaca, NY 14853, USA; Department of Botany, University of Wisconsin–Madison, Madison, WI 53706, USA; Negaunee Institute for Plant Conservation Science and Action, Chicago Botanic Garden, Glencoe, IL 60022, USA; Plant Biology and Conservation Program, Northwestern University, Evanston, IL 60208, USA; School of Integrative Plant Science, Section of Plant Biology and the L.H. Bailey Hortorium, Cornell University, Ithaca, NY 14853, USA; Department of Botany, University of Wisconsin–Madison, Madison, WI 53706, USA; Negaunee Institute for Plant Conservation Science and Action, Chicago Botanic Garden, Glencoe, IL 60022, USA; Plant Biology and Conservation Program, Northwestern University, Evanston, IL 60208, USA; School of Integrative Plant Science, Section of Plant Biology and the L.H. Bailey Hortorium, Cornell University, Ithaca, NY 14853, USA

**Keywords:** floral syndrome, genome annotation, HiC, Liliales, PacBio HiFi, scaffolding

## Abstract

*Calochortus tolmiei* Hook. & Arn., a bulbous monocot with cat's ear flowers in the angiosperm family Liliaceae, is a perennial herb native to northern California, Oregon, and Washington. *Calochortus* exhibits substantial morphological and karyotype diversity with multiple floral forms and a haploid chromosome number varying from 6 to 10. Here, we present the first high-quality reference assembly in Liliaceae for *C. tolmiei*, with a scaffolded assembly of 2.9 Gb with an N50 of 296 Mb. Notably, 92% of the assembled genome is scaffolded into 10 pseudomolecules, matching the documented chromosome count of *C. tolmiei.* The genome contains 31,049 protein-coding genes, with 86.2% being functionally annotated. The closest reference-quality genome assembly to *C. tolmiei* is from *Chionographis japonica* (Willd.) Maxim. (Melianthiaceae), which diverged ∼83 Mya, providing a valuable genomic resource in the Liliales, an order which lacks genomic resources.

## Introduction

The order Liliales contains ∼10 families, 64 genera, and ∼1,500 species, with the relationships among families fairly well understood based on plastome sequences (Givnish *et al.* 2016, 2018), yet relationships within major families remain controversial (Patterson and Givnish 2002; Fay *et al.* 2006; Lu *et al.* 2021). The Liliales is one of a handful of angiosperm clades that evolved giant genomes (>34 Gb; Leitch *et al.* 2007; Pellicer *et al.* 2014), most associated with a bulbous or geophytic habit, maintaining a large spread in 1C values ranging from 1.5 to 147 Gb (Leitch and Leitch 2013). Liliales includes many economically and horticulturally important species within the order such as lilies and tulips (*Lilium* and *Tulipa*; Marasek-Ciolakowska *et al.* 2018), fritillaries (*Fritillaria*; Day *et al.* 2014), and the source of the drug colchicine, the autumn crocus (*Colchicum autumnale*; Jung *et al.* 2011). Despite the importance of the order, the genomic resources within this group are sparse (Kress *et al.* 2022). Within the Liliales, the family Liliaceae is a taxonomically challenging and systemically important group (Badawi and Elwan 1986), consisting of ∼600 species with high levels of morphological diversity (Li *et al.* 2021). Within this biologically important family, there is a stark lack of reference-quality genome assemblies and annotations. The closest reference genome to any member of Liliaceae is *Chionographis japonica* (Willd.) Maxim. (Melanthiaceae; Kuo *et al.* 2023), which has a median divergence time of 83 million years from the focal taxon of this study, *Calochortus tolmiei* (https://timetree.org/; Kumar *et al.* 2017).

The genus *Calochortus* contains 74 species, all of them bulbous herbaceous plants, which range from the western United States into Mexico. *Calochortus* exhibits 4 floral “syndromes” (Ownbey 1940; Patterson and Givnish 2004), or suites of traits hypothesized to serve ecological functions such as pollinator attraction, herbivore exclusion, and regulation of water loss and temperature. These syndromes include (1) mariposa lilies, with large, brightly colored, upright flowers with a broad, cup-like form; (2) fairy lanterns (also known as globe lilies) with nodding flowers and a partly to completely closed, spherical flower formed by the valvate petals; (3) cat's ears, with open upright flowers with dense trichomes on the petals giving them a “hairy” appearance; and (4) star tulips with glabrous, triangular sepals, and petals held in a single plane. An updated phylogeny by Karimi *et al.* (2024) using Hyb-capture of 294 nuclear loci showed repeated evolution of these 4 floral syndromes, and multiple evolutionary transitions in chromosome number.

**Fig. 1.**
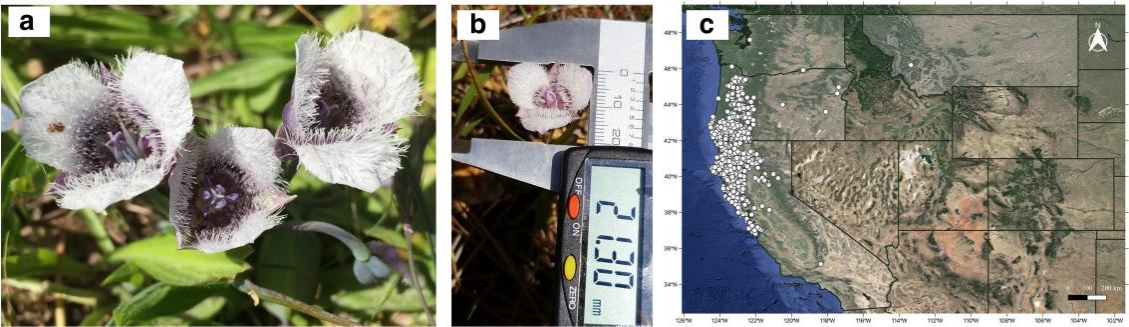
Morphology and distribution of *C. tolmiei*: a) Mature flowers, b) size of a single mature flower, and c) occurrence data from GBIF.org. Photo credit Chelsea D. Specht (a) and Evan Eiffler (b).

*Calochortus tolmiei*, a perennial herb, exhibits a cat's ear floral syndrome, with small flowers and white to purple petals densely covered in trichomes (Fig. 1a and b). The species is sister to a clade of 3 fairy-lantern species with yellow flowers (*Calochortus amabilis–Calochortus pulchellus–Calochortus rachei*) from the North Coast Ranges of California sister to a 4th cat's ear species (*Calochortus monophyllus*), also with yellow flowers, native to the northern Sierra Nevada (Karimi *et al.* 2024). *Calochortus tolmiei* is native to the western United States and restricted to Northern California, Oregon, and Washington (Ownbey 1940; Fig. 1c). The chromosome number of the species is 10 (Ownbey 1940; Beal and Ownbey 1943), which is conserved across closely related species in the “Bay Area Clade” (Patterson and Givnish 2004; Karimi *et al.* 2024), even though chromosome number is known to be highly variable within the genus and in close relatives (Peruzzi *et al.* 2009).

The goal of this study is to provide a reference genome and annotation for future work in *Calochortus* to investigate the genetic underpinnings for the repeated evolution of morphologies associated with different floral syndromes and shifts in chromosome number between closely related species, as well as provide the first reference-quality genome in the family Liliaceae and the second in the order Liliales. This new reference genome will enable researchers to address broad evolutionary questions across these ecologically, economically, and horticulturally important lineages of the Liliaceae and the Liliales.

## Methods

### Field collections and identification

Plants were collected in Butte County, California from 2 populations (39.72093N,121.51388W and 39.74421N,121.48878W) along public roads following local rules and regulations. Whole plants for both genome and transcriptome sequencing were shipped overnight to Cornell University wrapped in damp paper towels and stored at −80°C. Taxonomic identification was done by Evan Eifler following the Jepson Flora Project (2024). Herbarium accessions were deposited in the Wisconsin State Herbarium at the University of Wisconsin-Madison under vouchers v0405940WIS and v0405939WIS.

### Sequencing and genome assembly

Frozen tissue representing an entire individual including leaf and floral tissue was sent to Mount Sinai (New York, NY, USA) for DNA extraction and PacBio SMRTbell library prep for PacBio HiFi on the Sequel II 8M sequencing. In total, 4 single molecule, real-time (SMRT) cells were sequenced. Frozen tissue from a second individual, again an entire plant including leaf and floral tissue, was sent to Phase Genomics (Seattle, WA, USA) for HiC library construction and sequencing targeting 300 million read-pairs of sequencing (90 Gb) on the Illumina NovaSeq6000. Adapters and low-quality bases were trimmed from the raw Illumina sequencing files with fastp v0.23.4 (Chen *et al.* 2018) using default detection of adapters, a minimum quality score of 20, a minimum length of 75 bp, and trimming strings of poly-Gs. HiFiAdapterFilter (Sim *et al.* 2022) was used to filter out adapter sequences in the HiFi data with a minimum length of 44 bp and at least a 97% match. Transcriptome short-read (Illumina) data were generated from frozen floral tissue of *C. tolmiei* and closely related species (*Calochortus albus*, *Calochortus amabilis*, *Calochortus monophyllus*, and *Calochortus umbellatus*) at different floral bud stages, while long-read (Nanopore) data were generated from *Calochortus venustus*. Briefly, extracted RNA was sent to the UC Berkeley QB3 sequencing core (Berkeley, CA, USA) to build sequencing libraries by ribodepletion that were sequenced on the Illumina NovaSeq6000 2 × 150 (Illumina, San Diego, CA, USA) targeting ∼25 million reads per sample. Long-reads were generated using the cDNA-PCR Barcoding library kit (SQK-PCS109 with SQK-PBK004 barcodes) from 4 flower buds of the close relative.

HiFiasm v0.19.8 (Cheng *et al.* 2021, 2022) was used to assemble the HiFi and HiC data using the parameters -a 8 -l 3 –primary –dual-scaf. The primary genome assembly and both haplotypes were polished with racon v1.5.0 (https://github.com/isovic/racon) after mapping the HiFi reads to each assembly using minimap2 v2.27 (Li 2018) with the -x map-hifi option. Contaminants were removed from the polished assemblies using seqclean x86_64 (https://sourceforge.net/projects/seqclean/) using the eupathDB (Lu and Salzberg 2018) and UniVec (https://ftp.ncbi.nlm.nih.gov/pub/UniVec/) sequences. The cleaned assemblies were scaffolded with yahs v1.2 (Zhou *et al.* 2023) after mapping the clean HiC data with bwa-mem2 v2.2.1 (Vasimuddin *et al.* 2019) using default parameters. The resulting files were converted to binary alignment map (BAM) and sorted with samtools v1.19.2 (Li *et al.* 2009). Duplicate reads were marked using picard (http://broadinstitute.github.io/picard/) bundled with GATK v4.2.3.0 (McKenna *et al.* 2010) as recommended by yahs with the –ASSUME_SORT_ORDER queryname flag. The primary yahs commands for scaffolding were -e GATC,GANTC,CTNAG,TTAA with different resolution settings tested, and the value generating the most contiguous and fewest number of scaffolds was -r 5000,10000,20000,50000,100000,200000,500000,600000,700000,800000,900000. A HiC contact map for the primary scaffolded assembly was generated using the yahs recommendations and juicer tools v1.9.9 (Durand *et al.* 2016). After scaffolding, the assembly was checked for known contaminants using the foreign contaminant screener (FCS)-adaptor and FCS-GX (Astashyn *et al.* 2024) with 14 contigs each <70 kb being classified entirely as contaminants.

The quality of each of the genome assemblies was assessed with BUSCO v5.5.0 (Manni *et al.* 2021) using the liliopsida odb library, both pre- and post-scaffolding. The LTR Assembly Index (LAI) was calculated using LTR_retriever v.2.9.9 (Ou *et al.* 2018) following the recommended parameters on Github (https://github.com/oushujun/LTR_retriever). The newly proposed proportional N50 metric (Hjelmen 2024) and Quast (Gurevich *et al.* 2013) were not incorporated since there are no other reference genomes in the Liliaceae with which to make comparisons; as noted above, the closest relative with a reference-quality genome available is *Ch. japonica*, with an estimated divergence time of 83 Mya within the Liliales. However, the proportional N50 metric using the proportional difference of the estimated genome size and assembly size [(genome size − assembly size)/(genome size)] was calculated for the primary assembly and the 10 pseudomolecules of the primary assembly after scaffolding. Genome size estimation was initially done with a *k*-mer-based method using 21-mers with Jellyfish 2.3.1 (Marçais and Kingsford 2011) with the HiFi data; *k*-mer profiles were visualized with GenomeScope v2.0 and RESPECT (Sarmashghi *et al.* 2021). These estimates either failed to converge on a genome size prediction or estimated the genome to be larger than hypothesized. In the end, ModEst in the package backmap v0.5 (Pfenninger *et al.* 2022) was used to estimate genome size using the primary assembly and the scaffolded haplotype assembly.

EarlGrey v4.3.0 (Baril *et al.* 2024a, 2024b) was used for repeat annotation and masking after removing contigs <3 kb in length following the Braker manual recommendation. Illumina RNA-Seq reads for *C. tolmiei* and other *Calochortus* species (*C. albus*, *C. amabilis*, *C. monophyllus*, and *C. umbellatus*) were mapped to the soft-masked genome using hisat2 v2.2.1 (Kim *et al.* 2019) with the incorporation of read group information. Long-read Nanopore RNA-Seq from *C. venustus* was mapped to the soft-masked genome using minmap2 v2.27 (Li 2018) using -a -x splice -u f. The resulting files were converted to BAM (Binary Alignment Map) and sorted with samtools 1.19.2 (Li *et al.* 2009). Braker3 (Gabriel *et al.* 2024) was used for annotation of the scaffolded primary assemblies and the 2 haplotype assemblies with the mapped RNA-Seq data as well as protein sequences from the Viridiplantae OrthoDB using the following flags –AUGUSTUS_ab_initio –gff3. The completeness of the genome annotations was checked with BUSCO v5.5.0 (Manni *et al.* 2021) and the liliospdia odb10 library using the proteins mode and the amino acid file produced by Braker3. Annotated coding genes were functionally annotated by comparing against the Uniprot/Swissprot, GO, and KEGG databases using the nucleotide sequences and blastx v.2.13.0 (Camacho *et al.* 2009), the protein sequences and blastp v2.13.0 (Camacho *et al.* 2009), and HMMER v3.4 (hmmer.org).

### Distribution map

The species distribution map was generated using QGIS 3.34.6-Prizren (https://qgis.org/en/site/). Initially, the “world map” and Google satellite map layers were loaded into the QGIS workspace and utilized to delineate the borders of each state in the USA and visualize the satellite imagery of its surrounding regions, respectively. The “world map” layer was then clipped to display the geographic boundaries of Washington, Oregon, California, and Montana. Occurrence data from GBIF.org, with filtering settings of country to USA and “OccurrenceStatus” to present, were overlaid onto the clipped “world map” layer.

## Results and discussion

### Data generation and assembly

In total, 69 Gb of HiFi data were generated by Mount Sinai with a read length N50 of 8,259 bp and a mean read quality of 44.9. For the HiC data, 97 Gb (323,838,679 read-pairs) were generated by Phase Genomics. The combined assembly of the HiFi and HiC data generated a primary assembly of 2,420 contigs, with an N50 of 15 Mb, and a total assembled size of 2.9 Gb (2,916,850,758 bp) (Table 1). The 2 assembled haplotypes were less contiguous than the primary assembly, consisting of 3,442 contigs with an N50 of 3 Mb and a total assembly size of 2.9 Gb or 2,171 contigs an N50 of 4 Mb and an assembly size of 2.43 Gb. This discrepancy in size is not unexpected given that the primary assembly represents a combination of homologous haplotypes. Since some duplications are likely to exist in the primary assembly, we set the purge setting to the highest option (−l 3) which has been shown to be more reliable in removing duplicates than other existing tools (Cheng *et al.* 2022). In terms of completeness, the primary assembly and the haplotypes all had >90% complete BUSCOs with similar levels of duplications. The primary assembly was the best with 92.6% complete BUSCOs (56.7% being single copy and 35.9% being duplicated), 6.1% fragmented BUSCOs, and 1.3% missing out of the total 3,236 loci in the liliopsisda odb10 library. The 2 haplotypes were similar with 92.3% complete (58.7% single copy and 33.6% duplicated) and 90.2% complete (61.7% single copy and 28.5% duplicated), respectively. In comparison, the available genome of *Ch. japonica* (Kuo *et al.* 2023) showed 92.1% complete BUSCOs (84.7% single copy and 7.4% duplicated, 6.1% fragmented, and 1.8% missing).

**Table 1.** Assembly and scaffolding stats from the primary assembly and the 2 haplotypes, including size and BUSCO completeness using the liliopsida odb 10 library.

	Primary	Haplotype 1	Haplotype 2
Number of Contigs	2,420	3,442	2,171
Contig N50	15 Mb	3 Mb	4 Mb
Assembly size	2.9 Gb	2.9 Gb	2.43 Gb
BUSCO	C:92.1% [S:57.7%, D:34.4%], F:6.3%, M:1.6%	C:92.3% [S:58.7%, D:33.6%], F:6.5%, M:1.2%	C:90.2% [S:61.7%, D:28.5%], F:6.1%, M:3.7%
Number of scaffolds	1,998	2,855	1,498
Scaffold N50	296 Mb	321 Mb	251 Mb
Scaffold total size	2,896,294,695	2,870,403,126	2,426,414,324
Percent gaps	0.003%	0.048%	0.046%
Assembly % in 10 chromosomes	92%	85%	88%
# annotated genes	30,993	29,667	28,480
BUSCO	C:93.1% [S:39.2%, D:53.9%], F:4.7%, M:2.2%	C:92.4% [S:39.9%, D:52.5%], F:4.9%, M:2.7%	C:90.7% [S:42.4%, D:48.3%], F:5.0%, M:4.3%

For the BUSCO results abbreviations are C, complete BUSCOs; S, single copy; D, duplicated; F, fragmented; and M, missing.

**Fig. 2.**
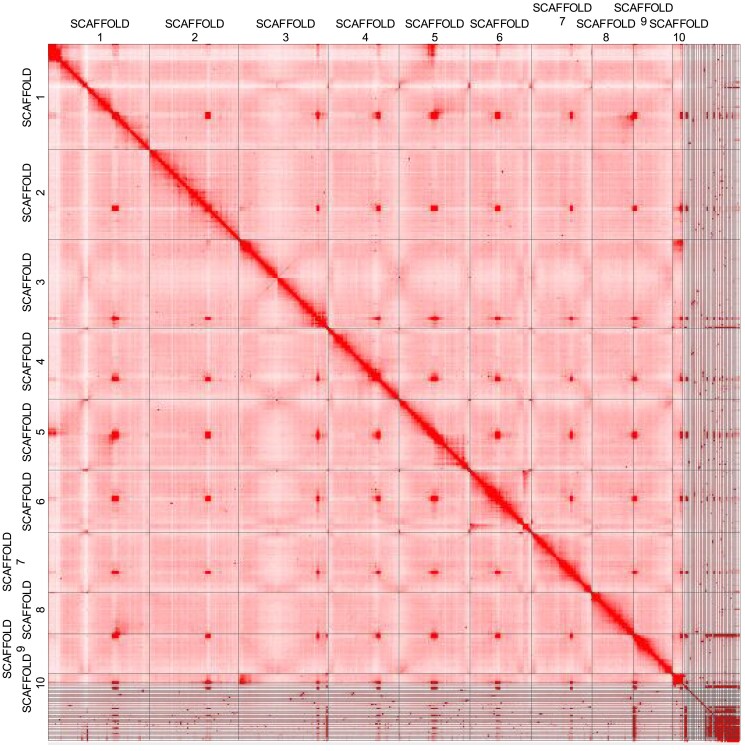
HiC contact map using the HiFiasm primary assembly scaffolded with YaHS displaying the 10 pseudomolecule scaffolds.

Scaffolding with yahs resulted in a primary assembly of 1,880 scaffolds (minimum of 3,000 bp in length) with an N50 of 296 Mb. Notably, 92% of the assembly was scaffolded into 10 pseudomolecules, matching the expected number of chromosomes in *C. tolmiei* (Ownbey 1940; Beal and Ownbey 1943). The longest pseudomolecule is 441,629,385 bp, while the shortest of the 10 pseudomolecules is 42,152,711 bp. The remaining 234 Mb of the assembly are in scaffolds under 8.5 Mb, with 133 scaffolds (198 Kb) removed prior to size calculation and annotation since they were under 3,000 bp. Species within many genera of Liliaceae (e.g. *Erythronium*, *Lilium*, and *Tulipa*) have long chromosomes and asymmetrical karyotypes (Sen 1975), yet no similarity in chromosome size or number has been found between *Calochortus* and related genera such as *Erythronium* or *Fritillaria* (Cave 1941). Given that only large-scale structures are of interest here (i.e. chromosomes), the lower resolution values in yahs were considered to be more appropriate (Lajoie *et al.* 2015). The HiC contact map shows a high level of contiguity in the pseudomolecules (Fig. 2).

### Genome assembly quality and content

The estimated genome size of *C. tolmiei* is 2.79 Gb using the HiFi data and the scaffolded haplotype assemblies with a peak coverage of 24× as calculated by MoDest. Using just the HiFi data and Genomescope or RESPECT generated inconsistent results with estimates of 1.7 and 4.5 Gb, respectively. The proportional genome size of the scaffolded primary assembly is −0.041 (assembly is larger than the estimated genome size), and when including just the 10 pseudomolecules the proportional difference is 0.043. This metric shows that the *C. tolmiei* assembly is within ∼4% of the estimated size, which is closer than most of the Viridiplantae genomes analyzed by Hjelmen (2024). The LAI of the scaffolded primary assembly was 15.28, which fits into the reference-quality genome category (Draft: 0 ≤ LAI < 10; Reference: 10 ≤ LAI < 20; Gold: 20 ≤ LAI; Ou *et al.* 2018).

**Fig. 3.**
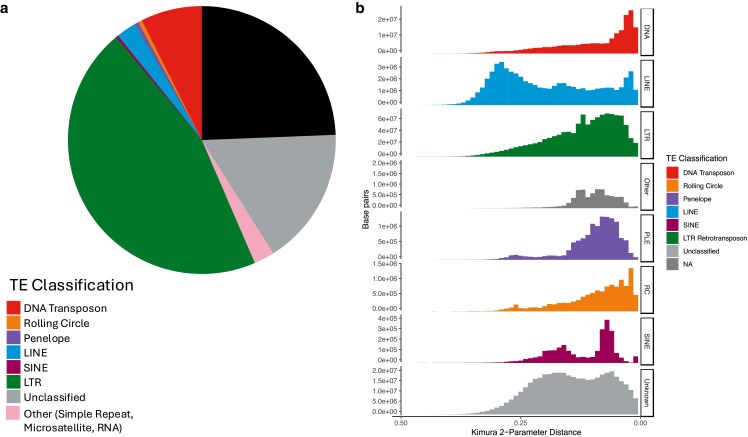
Repeat summary of the *C. tolmiei* genome including a) proportion of repeats, and b) repeat landscape distances split by repeat class.

Repeat masking with EarlGrey found that 24.1% of the *C. tolmiei* is composed of nonrepeat DNA, with the largest proportion of repeats being 42.5% LTR, 16.5% being unclassified repeats, DNA transposon being 7.3%, and the remaining repeat types making up a smaller percentage of the genome (Table 2; Fig. 3). *Chionographis japonica*, with an estimated genome size of 1.49 Gb (Pellicer and Leitch 2020) and assembly of 1.52 Gb (Kuo *et al.* 2023), in comparison, is composed of 15% nonrepeat DNA, 39.2% LTR, 18.9% simple repeats, 16.6% unclassified repeats, and 3.5% DNA transposons. The proportion of simple repeats (2.5 vs 18.9%) is one of the more striking differences between the 2 reference-quality assemblies. These notable differences in repeat content are supported by previous observations that large genomes in Liliaceae (e.g. *Fritillaria*) are not due to amplification of a few repeat families but highly heterogeneous, low-abundance repeats (Ambrozová *et al.* 2011; Lee and Kim 2014; Kelly *et al.* 2015), with species possessing smaller genomes having a more aggressive approach to removing young LTR retrotransposons (Michael 2014). Additional reference-quality genomes across the Liliaceae are needed to further investigate the nature of repeats across the family and look for patterns in the Liliales.

**Table 2.** Repeat classification of the *C. tolmiei* genome calculated by EarlGrey.

Repeat classification	Coverage	Count	Proportion	Number of distinct classifications
DNA	212,756,948	255,300	0.073	211
LINE	87,596,574	154,006	0.03	100
LTR	1,231,714,866	822,728	0.425	766
Other (Simple Repeat, Microsatellite, RNA)	173,107,576	44,926	0.060	45
Penelope	1,260,417	1,869	0.001	2
Rolling circle	11,467,012	20,769	0.004	22
SINE	3,194,890	7,281	0.001	6
Unclassified	478,155,422	1,083,332	0.165	918

Braker3 annotated 30,993 protein-coding genes in the scaffolded primary assembly with a BUSCO score of 93.1% complete (39.2% single copy and 53.9% duplicated), 4.7% fragmented, and 2.2% missing. The higher duplicated value in the annotation compared to the genome assembly is due to multiple isoforms in many of the genes. When only one isoform per gene was retained, the BUSCO score was 90% complete (56.8% single copy and 33.2% duplicated), 6.4% fragmented, and 3.6% missing, which more aligns with the genome scores (92.1, 57.7, 34.4, 6.3, and 1.6%, respectively; Table 1). The coding genes were annotated against the Uniprot/SwissProt, GO, and KEGG databases with 86.3% being functionally annotated.

### Conclusion

Here we report the first reference genome for *C. tolmiei*, assembled from PacBio HiFi and HiC data. This is the first reference-quality Liliaceae genome, and the second reference-quality assembly for the Liliales. This reference-quality genome will facilitate evolutionary studies in changes of floral syndromes and chromosome numbers between closely related species of *Calochortus*.

## Data Availability

The genome data generated have been deposited in the NCBI database under BioProject PRJNA1157915). PacBio HiFi data have been deposited in the Sequence Read Archive with accession SRR30586400, the HiC data with accession SRR30586399; the Illumina RNA-Seq data used for genome annotations are deposited under BioProject PRJNA1158208 with accessions SRR30588915–SRR30588921. The primary genome assembly and the haplotype assemblies have been deposited in GSA FigShare (https://doi.org/10.25387/g3.28057373). The primary assembly has been deposited at GenBank under the accession JBKFYM000000000. The version described in this paper is version JBKFYM010000000.

## References

[jkaf008-B1] Baril T, Galbraith J, Hayward A. 2024a. Earl Grey. Zenodo. 10.1093/molbev/msae068 PMC11003543 38577785

[jkaf008-B2] Ambrozová K, Mandáková T, Bures P, Neumann P, Leitch IJ, Koblízková A, Macas J, Lysak MA. 2011. Diverse retrotransposon families and an AT-rich satellite DNA revealed in giant genomes of *Fritillaria* lilies. Ann Bot. 107:255–268. 10.1093/aob/mcq235. 21156758 PMC3025733

[jkaf008-B3] Astashyn A, Tvedte ES, Sweeney D, Sapojnikov V, Bouk N, Joukov V, Mozes E, Strope PK, Sylla PM, Wagner L, et al 2024. Rapid and sensitive detection of genome contamination at scale with FCS-GX. Genome Biol. 25:60. 10.1186/s13059-024-03198-7. 38409096 PMC10898089

[jkaf008-B4] Badawi AA, Elwan Z. 1986. A taxonomic study of Liliaceae sensu lato. I. & II. Phytologia. 60:201–221.

[jkaf008-B5] Baril T, Galbraith J, Hayward A. 2024b. Earl grey: a fully automated user-friendly transposable element annotation and analysis pipeline. Mol Biol Evol. 41:msae068. 10.1093/molbev/msae068. 38577785 PMC11003543

[jkaf008-B6] Beal JM, Ownbey M. 1943. Cytological studies in relation to the classification of the genus *Calochortus*. III. Bot Gaz. 104:553–562. 10.1086/335169.

[jkaf008-B7] Camacho C, Coulouris G, Avagyan V, Ma N, Papadopoulos J, Bealer K, Madden TL. 2009. BLAST+: architecture and applications. BMC Bioinformatics. 10:421. 10.1186/1471-2105-10-421. 20003500 PMC2803857

[jkaf008-B8] Cave MS . 1941. Megasporogenesis and embryo sac development in *Calochortus*. Am J Bot. 28:390. 10.1002/j.1537-2197.1941.tb07985.x.

[jkaf008-B9] Chen S, Zhou Y, Chen Y, Gu J. 2018. Fastp: an ultra-fast all-in-one FASTQ preprocessor. Bioinformatics. 34:i884–i890. 10.1093/bioinformatics/bty560. 30423086 PMC6129281

[jkaf008-B10] Cheng H, Concepcion GT, Feng X, Zhang H, Li H. 2021. Haplotype-resolved *de novo* assembly using phased assembly graphs with hifiasm. Nat Methods. 18:170–175. 10.1038/s41592-020-01056-5. 33526886 PMC7961889

[jkaf008-B11] Cheng H, Jarvis ED, Fedrigo O, Koepfli K-P, Urban L, Gemmell NJ, Li H. 2022. Haplotype-resolved assembly of diploid genomes without parental data. Nat Biotechnol. 40:1332–1335. 10.1038/s41587-022-01261-x. 35332338 PMC9464699

[jkaf008-B12] Day PD, Berger M, Hill L, Fay MF, Leitch AR, Leitch IJ, Kelly LJ. 2014. Evolutionary relationships in the medicinally important genus *Fritillaria* L. (Liliaceae). Mol Phylogenet Evol. 80:11–19. 10.1016/j.ympev.2014.07.024. 25124097

[jkaf008-B13] Durand NC, Shamim MS, Machol I, Rao SSP, Huntley MH, Lander ES, Aiden EL. 2016. Juicer provides a one-click system for analyzing loop-resolution Hi-C experiments. Cell Syst. 3:95–98. 10.1016/j.cels.2016.07.002. 27467249 PMC5846465

[jkaf008-B14] Fay MF, Chase MW, Rønsted N, Devey DS, Pillon Y, Pires JC, Petersen G, Seberg O, Davis JI. 2006. Phylogenetics of Liliales: summarized evidence from combined analyses of five plastid and one mitochondrial loci. Aliso. 22:559–565. 10.1111/jbi.12486.

[jkaf008-B15] Gabriel L, Brůna T, Hoff KJ, Ebel M, Lomsadze A, Brodovsky M, Stanke M. 2024. BRAKER3: Fully automated genome annotation using RNA-seq and protein evidence with GeneMark-ETP, AUGUSTUS and TSEBRA. bioRxiv 544449. 10.1101/2023.06.10.544449, preprint: not peer reviewed. PMC11216308 38866550

[jkaf008-B16] Givnish TJ, Zuluaga A, Marques I, Lam VKY, Gomez MS, Iles WJD, Ames M, Spalink D, Moeller JR, Briggs BG, et al 2016. Phylogenomics and historical biogeography of the monocot order Liliales: out of Australia and through Antarctica. Cladistics. 32(6):581–605. 10.1111/cla.12153. 34727673

[jkaf008-B17] Givnish TJ, Zuluaga A, Spalink D, Soto Gomez M, Lam VKY, Saarela JM, Sass C, Iles WJD, de Sousa DJL, Leebens-Mack J, et al 2018. Monocot plastid phylogenomics, timeline, net rates of species diversification, the power of multi-gene analyses, and a functional model for the origin of monocots. Am J Bot. 105:1888–1910. 10.1002/ajb2.1178. 30368769

[jkaf008-B18] Gurevich A, Saveliev V, Vyahhi N, Tesler G. 2013. QUAST: quality assessment tool for genome assemblies. Bioinformatics. 29:1072–1075. 10.1093/bioinformatics/btt086. 23422339 PMC3624806

[jkaf008-B19] Hjelmen CE . 2024. Genome size and chromosome number are critical metrics for accurate genome assembly assessment in Eukaryota. Genetics. 227:iyae099. 10.1093/genetics/iyae099. 38869251

[jkaf008-B20] Jepson Flora Project . 2024. *Jepson eFlora*. https://ucjeps.berkeley.edu/eflora/. Accessed October 1, 2024.

[jkaf008-B21] Jung LS, Winter S, Eckstein RL, Kriechbaum M, Karrer G, Welk E, Elsässer M, Donath TW, Otte A. 2011. *Colchicum autumnale* L. Perspect Plant Ecol Evol Syst. 13:227–244. 10.1016/j.ppees.2011.04.001.

[jkaf008-B22] Karimi N, Krieg CP, Spalink D, Lemmon AR, Lemmon EM, Eifler E, Hernández AI, Chan PW, Rodríguez A, Landis JB, et al 2024. Chromosomal evolution, environmental heterogeneity, and migration drive spatial patterns of species richness in *Calochortus* (Liliaceae). Proc Natl Acad Sci U S A. 121:e2305228121. 10.1073/pnas.2305228121. 38394215 PMC10927571

[jkaf008-B23] Kelly LJ, Renny-Byfield S, Pellicer J, Macas J, Novák P, Neumann P, Lysak MA, Day PD, Berger M, Fay MF, et al 2015. Analysis of the giant genomes of *Fritillaria* (Liliaceae) indicates that a lack of DNA removal characterizes extreme expansions in genome size. New Phytol. 208:596–607. 10.1111/nph.13471. 26061193 PMC4744688

[jkaf008-B24] Kim D, Paggi JM, Park C, Bennett C, Salzberg SL. 2019. Graph-based genome alignment and genotyping with HISAT2 and HISAT-genotype. Nat Biotechnol. 37:907–915. 10.1038/s41587-019-0201-4. 31375807 PMC7605509

[jkaf008-B25] Kress WJ, Soltis DE, Kersey PJ, Wegrzyn JL, Leebens-Mack JH, Gostel MR, Liu X, Soltis PS. 2022. Green plant genomes: what we know in an era of rapidly expanding opportunities. Proc Natl Acad Sci U S A. 119:e2115640118. 10.1073/pnas.2115640118. 35042803 PMC8795535

[jkaf008-B26] Kumar S, Stecher G, Suleski M, Hedges SB. 2017. TimeTree: a resource for timelines, timetrees, and divergence times. Mol Biol Evol. 34:1812–1819. 10.1093/molbev/msx116. 28387841

[jkaf008-B27] Kuo Y-T, Câmara AS, Schubert V, Neumann P, Macas J, Melzer M, Chen J, Fuchs J, Abel S, Klocke E, et al 2023. Holocentromeres can consist of merely a few megabase-sized satellite arrays. Nat Commun. 14:3502. 10.1038/s41467-023-38922-7. 37311740 PMC10264360

[jkaf008-B28] Lajoie BR, Dekker J, Kaplan N. 2015. The Hitchhiker's guide to Hi-C analysis: practical guidelines. Methods. 72:65–75. 10.1016/j.ymeth.2014.10.031. 25448293 PMC4347522

[jkaf008-B29] Lee S-I, Kim N-S. 2014. Transposable elements and genome size variations in plants. Genomics Inform. 12:87–97. 10.5808/GI.2014.12.3.87. 25317107 PMC4196380

[jkaf008-B30] Leitch IJ, Beaulieu JM, Cheung K, Hanson L, Lysak MA, Fay MF. 2007. Punctuated genome size evolution in Liliaceae. J Evol Biol. 20:2296–2308. 10.1111/j.1420-9101.2007.01416.x. 17956392

[jkaf008-B31] Leitch IJ, Leitch AR. 2013. Genome size diversity and evolution in land plants. In: Greilhuber J, Dolezal J, Wendel J, editors. Plant genome diversity. Volume 2. Springer. p. 307–322.

[jkaf008-B32] Li H . 2018. Minimap2: pairwise alignment for nucleotide sequences. Bioinformatics. 34:3094–3100. 10.1093/bioinformatics/bty191. 29750242 PMC6137996

[jkaf008-B33] Li J, Cai J, Qin H-H, Price M, Zhang Z, Yu Yan, Xie D-F, He X-J, Zhou S-D, Gao X-F. 2021. Phylogeny, age, and evolution of tribe Lilieae (Liliaceae) based on whole plastid genomes. Front Plant Sci. 12:699226. 10.3389/fpls.2021.699226. 35178055 PMC8845482

[jkaf008-B34] Li H, Handsaker B, Wysoker A, Fennell T, Ruan J, Homer N, Marth G, Abecasis G, Durbin R. 2009. The sequence alignment/map format and SAMtools. Bioinformatics. 25:2078–2079. 10.1093/bioinformatics/btp352. 19505943 PMC2723002

[jkaf008-B35] Lu J, Salzberg SL. 2018. Removing contaminants from databases of draft genomes. PLoS Comput Biol. 14:e1006277. 10.1371/journal.pcbi.1006277. 29939994 PMC6034898

[jkaf008-B36] Lu R-S, Yang T, Chen Y, Wang S-Y, Cai M-Q, Cameron KM, Li P, Fu C-X. 2021. Comparative plastome genomics and phylogenetic analyses of Liliaceae. Bot J Linn Soc. 196:279–293. 10.1093/botlinnean/boaa109.

[jkaf008-B37] Manni M, Berkeley MR, Seppey M, Simão FA, Zdobnov EM. 2021. BUSCO update: novel and streamlined workflows along with broader and deeper phylogenetic coverage for scoring of eukaryotic, prokaryotic, and viral genomes. Mol Biol Evol. 38:4647–4654. 10.1093/molbev/msab199. 34320186 PMC8476166

[jkaf008-B38] Marasek-Ciolakowska A, Nishikawa T, Shea DJ, Okazaki K. 2018. Breeding of lilies and tulips-interspecific hybridization and genetic background. Breed Sci. 68:35–52. 10.1270/jsbbs.17097. 29681746 PMC5903980

[jkaf008-B39] Marçais G, Kingsford C. 2011. A fast, lock-free approach for efficient parallel counting of occurrences of k-mers. Bioinformatics. 27:764–770. 10.1093/bioinformatics/btr011. 21217122 PMC3051319

[jkaf008-B40] McKenna A, Hanna M, Banks E, Sivachenko A, Cibulskis K, Kernytsky A, Garimella K, Altshuler D, Gabriel S, Daly M, et al 2010. The genome analysis toolkit: a MapReduce framework for analyzing next-generation DNA sequencing data. Genome Res. 20:1297–1303. 10.1101/gr.107524.110. 20644199 PMC2928508

[jkaf008-B41] Michael TP . 2014. Plant genome size variation: bloating and purging DNA. Brief Funct Genomics. 13:308–317. 10.1093/bfgp/elu005. 24651721

[jkaf008-B42] Ou S, Chen J, Jiang N. 2018. Assessing genome assembly quality using the LTR assembly index (LAI). Nucleic Acids Res. 46:e126. 10.1093/nar/gky730. 30107434 PMC6265445

[jkaf008-B43] Ownbey M . 1940. A monograph of the genus *Calochortus*. Ann Mo Bot Gard. 27:371. 10.2307/2394384.

[jkaf008-B44] Patterson TB, Givnish TJ. 2002. Phylogeny, concerted convergence, and phylogenetic niche conservatism in the core Liliales: insights from rbcL and ndhF sequence data. Evolution. 56:233–252. 10.1111/j.0014-3820.2002.tb01334.x. 11926492

[jkaf008-B45] Patterson TB, Givnish TJ. 2004. Geographic cohesion, chromosomal evolution, parallel adaptive radiations, and consequent floral adaptations in *Calochortus* (Calochortaceae): evidence from a cpDNA phylogeny. New Phytol. 161:253–264. 10.1046/j.1469-8137.2003.00951.x.

[jkaf008-B46] Pellicer J, Kelly LJ, Leitch IJ, Zomlefer WB, Fay MF. 2014. A universe of dwarfs and giants: genome size and chromosome evolution in the monocot family Melanthiaceae. New Phytol. 201:1484–1497. 10.1111/nph.12617. 24299166

[jkaf008-B47] Pellicer J, Leitch IJ. 2020. The plant DNA C-values database (release 7.1): an updated online repository of plant genome size data for comparative studies. New Phytol. 226:301–305. 10.1111/nph.16261. 31608445

[jkaf008-B48] Peruzzi L, Leitch IJ, Caparelli KF. 2009. Chromosome diversity and evolution in Liliaceae. Ann Bot. 103:459–475. 10.1093/aob/mcn230. 19033282 PMC2707325

[jkaf008-B49] Pfenninger M, Schönnenbeck P, Schell T. 2022. ModEst: accurate estimation of genome size from next generation sequencing data. Mol Ecol Resour. 22:1454–1464. 10.1111/1755-0998.13570. 34882987

[jkaf008-B50] Sarmashghi S, Balaban M, Rachtman E, Touri B, Mirarab S, Bafna V. 2021. Estimating repeat spectra and genome length from low-coverage genome skims with RESPECT. PLoS Comput Biol. 17:e1009449. 10.1371/journal.pcbi.1009449. 34780468 PMC8629397

[jkaf008-B51] Sen S . 1975. Cytotaxonomy of Liliales. Feddes Repert. 86:255–305. 10.1002/fedr.19750860502.

[jkaf008-B52] Sim SB, Corpuz RL, Simmonds TJ, Geib SM. 2022. HiFiAdapterFilt, a memory efficient read processing pipeline, prevents occurrence of adapter sequence in PacBio HiFi reads and their negative impacts on genome assembly. BMC Genomics. 23:157. 10.1186/s12864-022-08375-1. 35193521 PMC8864876

[jkaf008-B53] Vasimuddin M, Misra S, Li H, Aluru S. 2019. Efficient architecture-aware acceleration of BWA-MEM for multicore systems. In: 2019 IEEE International Parallel and Distributed Processing Symposium (IPDPS); Brazil, IEEE. 314–324.

[jkaf008-B54] Zhou C, McCarthy SA, Durbin R. 2023. YaHS: yet another Hi-C scaffolding tool. Bioinformatics. 39:btac808. 10.1093/bioinformatics/btac808. 36525368 PMC9848053

